# Operator-Controlled Comparison Between 5 French and 6 French Guiding Catheters for Percutaneous Intervention Using Transradial Approach

**DOI:** 10.7759/cureus.8406

**Published:** 2020-06-02

**Authors:** Hisham Hirzallah, Kanaan Mansoor, Ahmed Amro, Julia Parsons, Mohamed S Suliman, Damir Kusmic, Yasir Jawaid, Yazan Numan, Sutoidem M Akpanudo, Ellen Thompson

**Affiliations:** 1 Cardiology, Marshall University Joan C. Edwards School of Medicine, Huntington, USA; 2 Internal Medicine, Marshall University Joan C. Edwards School of Medicine, Huntington, USA

**Keywords:** percutaneous coronary intervention, heart catheterization, 5 french catheters, 6 french catheters, transradial, radial access, contrast

## Abstract

Objective

To compare 5 French (Fr) and 6 Fr guiding catheters regarding the volume of contrast administered, fluoroscopy time, and total procedure time during transradial percutaneous coronary intervention (PCI).

Background

Previous studies had compared 5 Fr and 6 Fr catheters and deemed 5 Fr catheters safe and effective. In this study, we retrospectively compared the 5 Fr catheter to 6 Fr catheter with an attempt to eliminate the effect of inter-operator skill level variability.

Methods

In a single-center, retrospective cohort study, we randomly selected patients who had received PCI through transradial access using 5 Fr or 6 Fr catheters. The study involved two groups of 100 patients each. These groups were comprised of an equal number of cases from each operator. The primary endpoint was contrast medium volume. Secondary endpoints were fluoroscopy time and procedure time.

Results

Less contrast was used in the 5 Fr group vs. 6 Fr catheter group (140.2 ± 45.7 mL vs. 158.2 ± 66.7 mL, p=0.004). PCI using 5 Fr catheters was associated with shorter fluoroscopy time (13.7 ± 7.3 mins vs. 15.2 ± 8.2 mins, p=0.584) and shorter procedure time (43.7 ± 22.2 mins vs. 46.5 ± 19.7 mins, p=0.890), but this was not statistically significant.

Conclusion

Transradial PCI using 5 Fr guiding catheters was associated with less contrast medium usage, but there was no advantage regarding procedure time or fluoroscopy time when compared to 6 Fr catheters. Similar to 6 Fr catheters, 5 Fr catheters achieved high PCI success rates through radial access when compared in the treatment of coronary lesions with the same level of complexity.

## Introduction

Since its inception in 1978, the field of percutaneous coronary intervention (PCI) has been evolving to constantly better itself [1]. Constant innovation in the field has led to a decrease in complication rates and improvement in the efficacy of the procedures. Much of the innovation has been directed towards the development of new equipment, specifically guiding catheters. Initially, 9-10 French (Fr) catheters were used for procedures; however, there was a gradual reduction in the size of catheters, and, eventually, 6 Fr catheters were established as the standard of care. In 1989, a 5 Fr catheter was utilized for a coronary angioplasty for the first time.

Among the numerous medical devices manufactured, only a few make the cut and are deemed to be the standard of care after being studied and tested in the field. Before the 6 Fr catheter was determined to be the standard of care, it had also been compared and tested against 7 Fr and 8 Fr catheters; and it was crowned as the standard of care only after it was deemed to be effective [2-4]. With the advent of the 5 Fr catheter, a similar approach was undertaken before its utility was established in common practice. The question that arises with the advent of every new generation of modern catheters is whether or not it is as effective as its predecessor, particularly regarding its ability to deliver the necessary components such as balloons and stents during PCI. In this study, we retrospectively compared the 5 Fr catheter to its predecessor, the 6 Fr catheter, with an attempt to eliminate the effect of inter-operator skill level variability.

## Materials and methods

Study design and patient population

**Figure 1:**
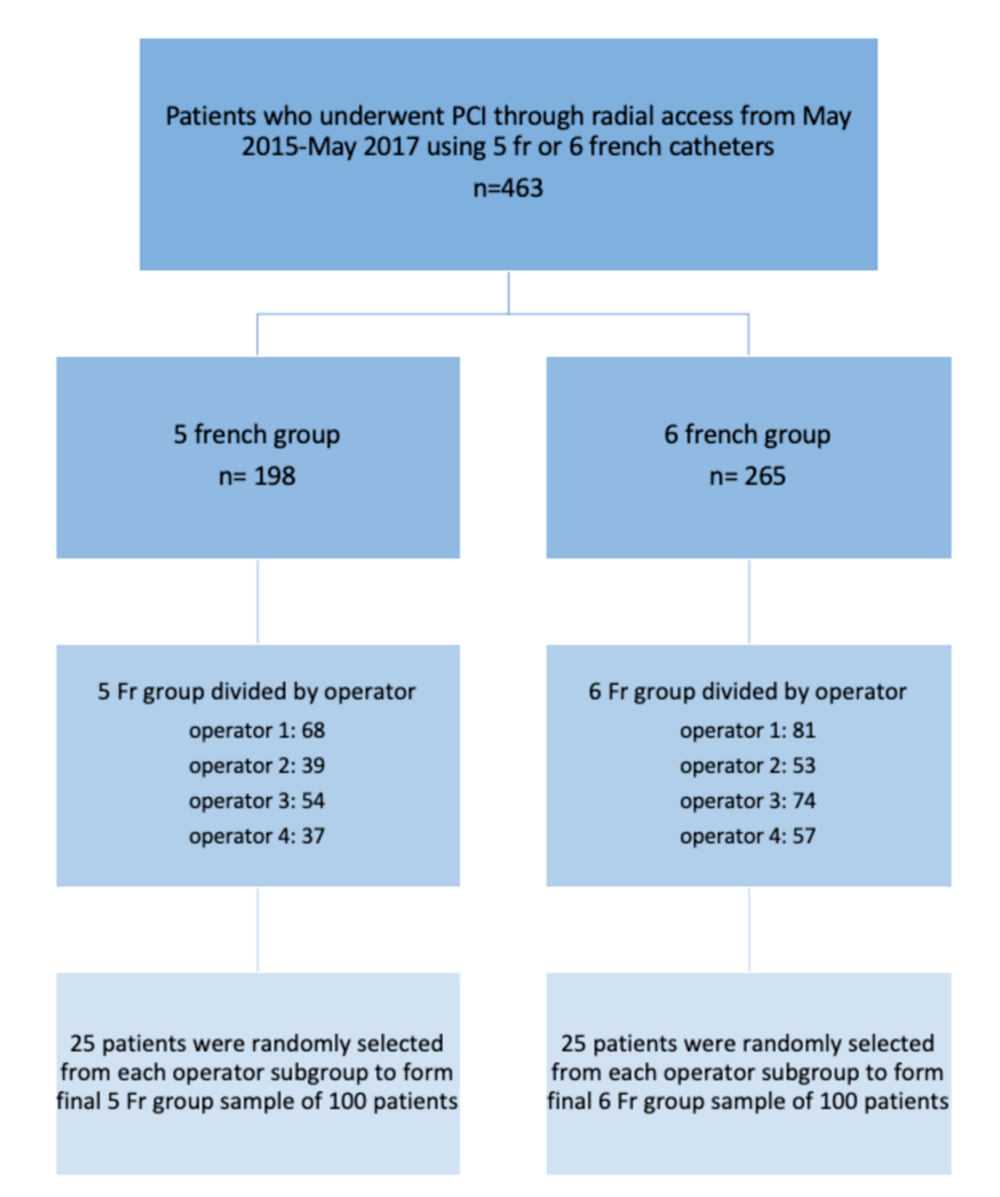
Patient population PCI: percutaneous coronary intervention

Our study was a single-center, retrospective cohort study designed to compare procedural variables of 5 Fr versus 6 Fr catheters in patients who underwent PCI using radial access. A total of 200 patients were involved in the retrospective analysis in this study. The study population was randomly selected from a pool of 463 patients that included all the consecutive PCI cases performed at St. Mary’s Medical Center - Marshall University by four different interventional cardiologists with expertise in transradial procedures during May 2016-May 2017. All of the cases used radial access as initial access, and at least one stent was deployed using 5 Fr or 6 Fr as an initial catheter. Indications for the procedure were either elective for stable angina or emergent for non-ST-elevation myocardial infarction (NSTEMI) and ST-elevation myocardial infarction (STEMI). The total study population was then divided into two groups: 5 Fr group and 6 Fr group. Of the total 463 cases that we considered, 265 were performed using 6 Fr catheter and 198 using 5 Fr catheter. Each of these two groups was then divided into four subgroups based on the operator (Figure 1). Twenty-five cases were randomly selected from each operator to be included in the study, resulting in the final two groups having 100 patients each with an equal proportion from each operator.

Procedure description

The angioplasty procedures were performed using 5 Fr or 6 Fr catheters according to the operator preference and clinical judgment about the clinical condition of the patient, the coronary anatomy, and the approaching condition of the lesion. All patients were pretreated with aspirin 100-300 mg and were given 5000 IU of heparin as a bolus after the insertion of the arterial sheath. An additional dose of 2500 IU of heparin was given if the procedure lasted more than 90 mins. Balloon angioplasty followed by stent deployment was performed in all cases. At the end of each procedure, a transradial band was applied to achieve hemostasis. Procedural success was defined as a less than 50% residual stenosis with Thrombolysis in Myocardial Infarction (TIMI) grade 3 anterograde flow without the occurrence of complications such as death from any cause, urgent coronary bypass surgery, or acute myocardial infarction.

Data analysis

Baseline clinical characteristics were analyzed for all patients included in the study. Results were expressed as proportions or mean with standard deviation (SD). The primary endpoint was the amount of contrast used in the procedure. Secondary endpoints included procedure time and fluoroscopy time. All were continuous variables and they were assessed by t-test using SPSS Statistics version 24.0 (IBM, Armonk, NY). A p-value of <0.05 was considered statistically significant.

## Results

A total of 200 patients were included in the analysis, with 100 each from the two study groups. Both groups consisted of unselected randomized patients in routine clinical practice with a diverse clinical profile. The two groups were well balanced regarding baseline characteristics (Table 1).

**Table 1:** Demographic data and clinical characteristics SD: standard deviation

Characteristics	5 French group (n=100)	6 French group (n=100)
Males, n (%)	40 (40%)	77 (77%)
Females, n (%)	60 (60%)	23 (23%)
Age (years), mean ± SD	64 ± 11.7	64 ± 11.9
Hypertension, n (%)	82 (82%)	78 (78%)
Diabetes mellitus, n (%)	42 (42%)	40 (40%)
Chronic kidney disease, n (%)	9 (9%)	10 (10%)
Dyslipidemia, n (%)	70 (70%)	65 (65%)
Peripheral artery disease, n (%)	12 (12%)	8 (8%)
Current smoker, n (%)	45 (45%)	36 (36%)
Previous myocardial infarction, n (%)	11 (11%)	18 (18%)

The mean age was 64 years in both groups with SD of 11.7 years in the 5 Fr group and 11.9 years in the 6 Fr group. The 5 Fr group had 40% males while the 6 Fr group had 77% males. NSTEMI was the most common indication for the procedures with 82% in the 5 Fr group and 77% in the 6 Fr group (Table 2).

**Table 2:** Angiographic characteristics PCI: percutaneous coronary intervention; CABG: coronary artery bypass grafting; NSTEMI: non-ST-elevation myocardial infarction; STEMI: ST-elevation myocardial infarction; SD: standard deviation

Characteristics	5 French group (n=100)	6 French group (n=100)
Previous PCI, n (%)	39 (39%)	40 (40%)
Previous CABG, n (%)	27 (27%)	29 (29%)
Presentation:		
NSTEMI, n (%)	82 (82%)	77 (77%)
STEMI, n (%)	8 (8%)	5 (5%)
Stable angina, n (%)	10 (10%)	18 (18%)
Preprocedural aspirin treatment, n (%)	100 (100%)	100 (100%)
Preprocedural GPIIb/IIIa inhibitor treatment, n (%)	78 (78%)	69 (69%)
PCI for >1 vessel, n (%)	20 (20%)	24 (24%)
Number of stents deployed, mean ± SD	1.4 ± 1.8	1.3 ± 2.1
Stent length (mm), mean ± SD	14.8 ± 9.3	15.2 ± 8.4
Crossover from radial to femoral access, n (%)	1 (1%)	3 (3%)
Group crossover, n (%)	3 (3%)	2 (2%)
Success rate, (%)	99%	98%

Crossover from 5 Fr to 6 Fr occurred in three cases (3%) in the 5 Fr group, while crossover from 6 Fr to 5 Fr occurred in two cases (2%) in the 6 Fr group. As defined in the methods section, a 99% success rate was achieved in the 5 Fr group while 98% success rate was achieved in the 6 Fr group. Compared with transradial PCI using 6 Fr catheters, the 5 Fr group had lower contrast use (140.2 ± 45.7 mL vs. 158.2 ± 66.7 mL, p=0.004). PCI using 5 Fr catheters was associated with shorter fluoroscopy time (13.7 ± 7.3 mins vs. 15.2 ± 8.2 mins, p=0.584). Again, procedure time was less with the 5 Fr catheters (43.7 ± 22.2 mins vs. 46.5 ± 19.7 mins, p=0.890). The latter two findings were statistically insignificant.

## Discussion

Initial prototypes of the 5 Fr and 6 Fr catheters did not have a lumen large enough for an adequate stent insertion as the lumen was less than 0.058 inches; hence, these catheters could only be employed for diagnostic purposes [5-9]. It was not until Medtronic (Medtronic plc, Dublin, Ireland) introduced a catheter with a diameter of 0.058 inches that allowed stents and balloons to pass that the potential of 5 Fr catheters was realized [10].

The appeal of 5 Fr catheter came to light when several studies reported favorable prospects of utilization. In 2001, Schöbel et al. reported a success rate of up to 95% in 5 Fr catheters used in transfemoral access site depending on the type of the lesion. However, it was also reported that 5 Fr catheters had limitations in the application such as poor back-up support in long lesions as well as severe stenosis [10]. Gobeil et al. designed a single-arm mixed study of transradial and transfemoral PCI approach that confirmed that PCIs were technically feasible using a 5 Fr guiding catheter with a success rate of 95% [11].

The 5 French catheter use has strongly been associated with decreased usage of contrast volume. Schussler et al. studied 5 Fr catheters with transradial PCI in preselected cases where they were shown to be effective. They concluded that the usage of 5 Fr catheters reduces morbidity and the cost of the procedure since it required less amount of contrast medium and resulted in minimal complications [12]. Rakhit et al. compared the 5 Fr to 6 Fr catheter in transfemoral PCI and found that the 5 Fr approach is both feasible and safe with an acceptable outcome. He reported that although there does not appear to be a clear advantage in terms of vascular complications, there is a clear advantage in terms of reduced contrast medium usage with 5 Fr catheters. The study listed several circumstances when the 5 Fr would not be suitable, such as using cutting balloons for restenosis or kissing balloons for bifurcation lesions, but ultimately concluded that they can be effectively applied to routine interventional practice [13].

Gobeil et al. compared the 5 Fr and 6 Fr in transradial PCIs [14]. Their study had a similar design to our study, except that in our study, each operator contributed an equal amount of procedures to the total sample of each group, which eliminated the effect of inter-operator skill level differences. During this process, randomization was applied in an attempt to avoid selection bias. Contrast volume used during PCI plays an important role in the outcome of the procedure as increased contrast has been linked to increased complications including contrast-induced nephropathy. Thus, we decided to make the contrast volume our primary endpoint. Contrary to the results of Gobeil et al., our study showed that 5 Fr catheter use was associated with less contrast volume use, which was statistically significant [14]. Procedural data analysis from both groups showed a similar average number of lesions treated and the number of stents placed. This would negate the argument that less contrast usage in the 5 Fr group could be attributed to less complicated cases with less intervention. This could be utilized when patients with reduced kidney function require PCI. Similar to Gobeil et al., our data analysis did not show any statistically significant difference in terms of procedure time or fluoroscopy time between the two study groups.

The success of the procedure was defined as less than 50% residual stenosis with TIMI grade 3 anterograde flow without the occurrence of complications such as death from any cause, urgent coronary bypass surgery, or acute myocardial infarction. 5 Fr catheters were associated with a 99% success rate, which is higher than the 6 Fr group (98%). Previous studies have shown the superiority of the use of 5 Fr catheters in terms of success and complications rates; however, the success rate of 99% is significantly higher than the rate reported in older studies like Gobeil et al., which showed a success rate of 90% [14]. In recent years, the access site for PCI has been under debate. The transfemoral approach is recognized as the conventional approach. In the 1990s, the transradial approach was introduced as a means to decrease complications and thereby reduce the length of stay and cost [15]. Subsequently, transradial PCI was in fact shown to be superior to transfemoral PCI in reducing bleeding; however, it came with some caveats. Anatomical variations such as the smaller diameter of the radial artery, radial loops, and insufficient collateral flow are considered to be the main reasons for post-procedural complications [16]. 5 Fr catheters become more useful in such circumstances as they are less stiff than their predecessors, thus overcoming the anatomical aberrance of the radial access [14]. The mean radial diameter ranges between just 2.20 to 2.40 mm, making the 5 Fr catheter the optimal choice [17]. The sheath for the 6 Fr is 2.55 mm, making it an unfavorable option and a potential cause for post-procedural radial artery spasm [18].

Dahm et al. conducted a randomized study of 5 Fr vs. 6 Fr transradial PCI aiming to ascertain whether the 5 Fr guiding catheter has equivalent procedural success and complication rates as the 6 Fr. Their study showed that transradial PCI of non-complex lesions can be successfully performed with either 5 Fr or 6 Fr guiding catheters, with 5 Fr showing higher procedural success rates and lower vascular access complications in comparison to 6 Fr guiding catheter [19]. In this study, however, data about the contrast amounts or procedure/fluoroscopy times were not collected.

## Conclusions

Our findings revealed that transradial PCI using 5 Fr guiding catheters was associated with less contrast medium usage when compared to 6 Fr catheters. 5 Fr catheters would thus be the preferred choice for patients with reduced renal function or when minimizing the use of contrast medium is of concern. 5 Fr catheters did not have an advantage over 6 Fr catheters in terms of procedure time or fluoroscopy time. Similar to 6 Fr catheters, 5 Fr catheters can achieve a high PCI success rate through radial access when compared in the treatment of coronary lesions with the same level of complexity.
